# Familial dietary intervention in children with excess body weight and its impact on eating habits, anthropometric and biochemical parameters

**DOI:** 10.3389/fendo.2022.1034148

**Published:** 2022-12-01

**Authors:** Kamilla Strączek, Anita Horodnicka-Józwa, Justyna Szmit-Domagalska, Tomasz Jackowski, Krzysztof Safranow, Elżbieta Petriczko, Mieczysław Walczak

**Affiliations:** ^1^ Department of Paediatrics, Endocrinology, Diabetology, Metabolic Diseases and Cardiology of the Developmental Age, Pomeranian Medical University, Szczecin, Poland; ^2^ Department of Biochemistry and Medical Chemistry, Pomeranian Medical University, Szczecin, Poland

**Keywords:** childhood obesity, lipid profile, nutrition, behavioral intervention, glucose metabolism

## Abstract

**Introduction:**

Obesity is considered a civilisation disease which increases mortality and impairs quality of life, also among children and adolescents. The prevalence of overweight and obesity is steadily increasing in the developmental age population. Environmental factors are responsible for the main reason of excessive adipose tissue accumulation. Among these, poor eating habits and lack of exercise play the largest role. Familial prevalence of obesity and family dietary patterns also receive significant attention. Many specialists believe that the treatment of obesity should be multidirectional, effective and minimally invasive. Therefore, effective and safe methods are being investigated to effectively reduce body weight and improve eating habits. Dietary education programmes are an alternative to improve the health status of obese and overweight children and adolescents. To be fully effective, these programmes should involve the whole family.

**Aim of the study:**

In the face of constantly increasing prevalence of overweight and obesity in the developmental age population and the lack of effective methods to combat its occurrence, it seems appropriate to try to assess the effectiveness of a one-year-long dietary education of children and adolescents with excess body weight on their eating habits and the eating habits of their mothers, as well as selected anthropometric and biochemical parameters in these children using a simple educational tool, the Healthy Food Pyramid.

**Patients and methods:**

The study group consisted of 68 children with overweight and obesity, patients of the Department of Paediatrics, Endocrinology, Diabetology, Metabolic Diseases and Cardiology of the Developmental Age of the Pomeranian Medical University in Szczecin. The study used a proprietary questionnaire to assess dietary habits. Patients participated in six individual educational meetings over a twelve-month period. Eating habits were assessed in children and mothers before and after dietary intervention. Sixty-seven questionnaires before and after the dietary intervention were used for analysis.

**Results:**

Sixty-eight children completed the study. Those who did not complete the study came from families living in rural areas and their mothers mostly had primary or vocational education. One-year dietary education resulted in significant improvements in body weight, waist and hip circumference, WHtR and selected measured carbohydrate and lipid metabolism parameters with the exception of total cholesterol. The one-year dietary intervention did not have the same effect on the change in dietary habits in children and in their mothers.

## Introduction

Obesity has accompanied humans since prehistoric times. It was considered a sign of prosperity, successful social status and health (1). Nowadays, it is recognised as a civilisation disease that increases mortality and diminishes quality of life, also among children and adolescents (2). Among 5-19 year olds, the prevalence of overweight and obesity has increased dramatically from 4.0% to 22.0% over the last four decades (3, 4).

Overweight and obesity in childhood carry many health risks. It is a risk for the development of musculoskeletal disorders, type 2 diabetes, liver disease, as well as cardiovascular disease and emotional and psychosocial problems (5).

environmental factors are responsible for the main reason for the accumulation of excessive adipose tissue. Among these, poor dietary habits and lack of exercise play the greatest role (6). The family prevalence of obesity (7) and family dietary patterns (8) have also received considerable attention. The parents’ diet acts as a pattern for the child’s nutrition. Parents shape dietary habits, especially in the early years of a child’s life. The family diet determines what habits the child will adopt and how their diet will be shaped in adulthood (9). One of the most common incorrect dietary habits in overweight children is not eating breakfast regularly or skipping it altogether (10).

Additionally, it is worrying that the frequency of eating decreases with the age of the child (11). Another abnormal eating habit is snacking between meals, mainly on sweets, the consumption of which at least once a day was declared by as many as 60.0% of primary school children (12). Highly undesirable, and commonly reported among school children, is the consumption of fast-foods. Consumption of this type of food at least once a month was declared by 30.0% of 8–10 year olds (13). A very worrying trend is the increasing consumption of this type of food in the youngest age groups. After the age of 12 months, 2.5% of children ate this type of food at least once a month, and at pre-school age 11.5% at least once a week. In the same age group, eating two dinners is equally common. This results in overfeeding and a consequent imbalance in the body’s energy intake, which, in the absence of sufficient physical activity, results in increased body weight (14). A major controversy in terms of child and adolescent health is the amount of sweetened, carbonated and non-carbonated drinks consumed. Among 10–17 year olds, coloured fizzy drinks are listed as the second most consumed liquid of the day (15). It has also been shown that the incidence of overweight was higher in children who consumed more than 350 ml of juice per day (16). The habit of consuming sufficient amounts of fruit and vegetables is still unsatisfactory (17). Children mainly consume them in the form of salads with lunch (18) and only 1 in 10 preschool children meet the recommended intake of 4-5 portions of vegetables per day (19). Furthermore, the lack of acceptance of cereal products containing whole grains is worrying. Approximately 90.0 per cent of adolescents do not eat whole grain cereal products, such as whole grain bread, on a daily basis, and the consumption of groats or rice less than once a week was declared by almost 50.0 per cent of adolescents. Lack of the habit of eating fish is still a major problem. Their occasional consumption is declared by 50.0–70.0% of schoolchildren (20). The reluctance to consume fish does not change even in those with unrestricted access to it. Only less than 45.0% of adolescents from schools at the seaside meet WHO expert recommendations for regular consumption of fish at least 1–2 times a week (21). In addition to poor dietary habits, low physical activity is an important factor increasing the risk of obesity and overweight in children. In recent years, physical activity patterns of school-aged children, have changed dramatically. Industrialisation, technical progress and modern means of transport have created ideal conditions for the development of obesity. Television, computers, limited access to playgrounds and additional activities at school have contributed to a reduction in physical activity (22). On average, 5–10 hours of physical activity per week were declared by only about 28.0% 6–10 year olds, and about 27.0% reported only 3-5 hours of physical activity/week (23).

Many specialists believe that the treatment of obesity should be multidirectional, effective and, if possible, as minimally invasive as possible. In addition to the use of restrictive diets, pharmacotherapy and bariatric surgery, nutrition education programmes are an alternative to improve the health of obese and overweight children and adolescents. To be fully effective, these programmes should involve the whole family, or at least those most involved in perpetuating healthy eating patterns, usually the mother. One such model is the “Healthy Food Pyramid”, created by a group of experts from the Institute of Food and Nutrition in Warsaw. “The Healthy Food Pyramid” is a graphic illustration of the principles of proper nutrition, which forms the basis of dietary recommendations in Poland (24).

Considering the constant increase in the prevalence of overweight and obesity in the developmental age population and the lack of effective methods to combat its prevalence, it seems advisable to attempt to assess the effect of one-year-long dietary education of children and adolescents with excessive body weight on their eating habits and the eating habits of their mothers, as well as selected anthropometric and biochemical parameters in these children, using a simple educational tool, which is the Food Pyramid.

## Patients and methods

### Eligibility

Children aged 3–18 years and their parents, referred to the Department of Paediatrics were invited to the study for planned diagnostics of the causes of excessive body weight. Ultimately, only mothers participated in the study with their children.

Participation in the study was voluntary. Each mother and child over 13 years of age were given written information about the purpose of the study. Informed consent to participate in the study was obtained from each mother, as well as children over 13 years of age.

Approval for the study was obtained from the Bioethics Committee at the (decision number KB-0012/34/11, dated 16^th^ May 2011).

Children with underlying conditions were excluded, such as:

- congenital diseases predisposing to obesity, e.g. Down syndrome, Prader-Willi syndrome,
- thyroid disorders,
- adrenal gland dysfunction,
- gonadal disorders,
- intellectual disabilities,
- chronically ill children whose treatment may have influenced weight gain, such as steroid therapy.

Ninety-four children with excess body weight, The study was completed by 68 (72.3%) children, aged 4-17 years (x̅=12.4 ± 3.7), including 37 (54.4%) girls and 31 (45.6%) boys.

Anthropometric parameters, biochemical test and dietary habits were assessed in this group. All measurements were made before and after the dietary intervention.

### Methods of anthropometric measurements and adipose tissue measurement

The following anthropometric parameters were measured in the children: body height, with an accuracy of 0.01 cm, using a stadiometer (type Harpenden 602VR, UK); body weight, with an accuracy of 0.01 kg, on a medical scale (Radwag WPT 60/150 OW, Poland); waist circumference, with an accuracy of 0.5 cm, with a centimetre tape.

The obtained results of individual measurements were related to the norms for the population of Polish children, developed in the OLA and OLAF project (25, 26). The cut-off points defining overweight and obesity within this project are consistent with the criteria of the International Obesity Task Force (IOTF) (27, 28).

Based on the measurements taken, body mass index (BMI) was calculated, according to the formula: BMI = body weight (kg)/body height (m2). Waist-height ratio (WHtR), according to the formula: WHtR = waist circumference(cm)/body height (cm). Abdominal obesity was diagnosed when waist circumference was ≥90^th^ percentile for sex and age and WHtR >0.5 (29).

A standard deviation-score (SDS) was calculated to eliminate the effect of age and sex of the studied children on the measured anthropometric parameters. To calculate the SDS, reference values for the population of Polish children from the OLA and OLAF project were used (25, 26). According to the adopted criteria, BMI ≥+1SDS and <+2 SDS was considered overweight, and BMI ≥2 SDS was considered obesity. Body fat and lean muscle content were measured using an electrical bioimpedance analyser (Jawon IOI-353, Selvas Healthcare, South Korea). The test was performed according to the manufacturer’s instructions. As specified by the manufacturer, the test was not performed in children under 5 years of age.

### Methods of anthropometric measurements in mothers of the studied children

The data collected during the interview and available medical records were used to calculate the body mass index in the mothers of the studied children. BMI was calculated from these data. WHO criteria were used to assess the nutritional status of the mothers (30).

### Methods for biochemical measurements

Carbohydrate metabolism was assessed by measuring glucose and insulin levels at fasting and 120 min after an oral glucose tolerance test with load of 1.75 g glucose/kg body weight (maximum 75 g). Serum glucose and insulin concentrations were determined using Cobas C501 device (Roche, Germany).

For the assessment of carbohydrate metabolism, the guidelines of the Polish Diabetes Association (31) were used, assuming the following values for fasting measurements: normal fasting glycaemia: 70–99 mg/dl; impaired fasting glucose (IFG): 100–125 mg/dl; fasting glycaemia ≥126 mg/dl – diabetes mellitus. The following glycaemic values were assumed at 120 minutes of the OGTT: normal glucose tolerance (NGT): glycaemia <140 mg/dl; impaired glucose tolerance (IGT): glycaemia 140–199 mg/dl, diabetes mellitus: glycaemia ≥200 mg/dl.

Hyperinsulinaemia at 120 min of the OGTT test was diagnosed at insulin levels >75 µIU/ml (32).

To assess serum lipid metabolism in the study group, total cholesterol, LDL-cholesterol, HDL-cholesterol, triglyceride (TG) concentrations were measured using Cobas C501 device (Roche, Germany). The National Cholesterol Education Program (NCEP) expert guidelines (33) were used to assess lipid metabolism, adopting the following reference values: total cholesterol – <170 (mg/dl); LDL-cholesterol – <110 (mg/dl); HDL-cholesterol – >45 (mg/dl); triglycerides: <75 (mg/dl) (0–9 years of age); <90 (mg/dl) (10–19 years of age).

Using the measured biochemical parameters, HOMA insulin resistance index (HOMA-IR, Homeostasis Model Assessment – Insulin Resistance) was calculated, using the formula: HOMA-IR index= fasting glucose concentration (mg/dl) x fasting insulin concentration (µIU/ml)/405 (34).

The literature shows that there is no established value for determining insulin resistance in children. It has been shown that HOMA-IR increases during adolescence (35). Due to the heterogeneity of the study group in terms of age and sex, HOMA-IR centiles developed for the Caucasian population were used, and insulin resistance was diagnosed at HOMA-IR ≥97^th^ percentile for age and sex [33/39/].

### Diet analysis methods

The study used the dietary survey method and the research tool of a proprietary questionnaire to assess dietary habits and frequency of consumption. The nutrition part of the questionnaire was preceded by questions relating to the socio-demographic characteristics of the subjects and the anthropometric parameters of mothers and children. The dietary questionnaire for mother and child consisted of 30 questions. In the questions concerning frequency of product intake, a 7-point scale was used to assess it, where the answer, several times a day scored 6 points, once a day – 5 points, several times a week – 4 points, once a week – 3 points, several times a month – 2 points, once a month – 1 point, does not eat – 0 points, and a 3-point scale of answers, where the answers were scored: eats for every meal – 2 points, eats but not for every meal – 1 point, does not eat – 0 points. Children under 13 years of age completed the questionnaire with their mother, whereas children over 13 years of age completed the questionnaire on their own or with the mother’s help.

The “Healthy Food Pyramid” and the 10 Principles of Healthy Eating of Children and Adolescents developed by the Institute of Food and Nutrition in Warsaw, 2009, were used to change eating habits and improve nutrition (24). In education, particular emphasis was placed on: reducing the intake of saturated fat in the diet, by: eliminating snacks such as crisps, nuts, salty sticks and fast-foods from the diet, limiting fried meals in favour of stewed, boiled and steamed ones, increasing the consumption of fish and lean meat and dairy products; increasing the amount of fruit and vegetables consumed; reducing the intake of sugar, sweets and sweetened dairy products; eliminating sweetened drinks and fruit juices; introducing whole grain cereal products; drinking water daily and eating an adequate amount of food regularly; increasing control over nutritional behaviours and informed decisions considering choosing the right product – the ability to analyse the products’ labels; encouraging daily physical activity.

### Dietary education

During the study, children and their mothers participated in 6 individual educational meetings during which dietary education and correction of dietary errors were carried out. During the first 3 months, follow-ups took place at a frequency of 1 meeting per month, follow-up 4, 5 and 6 – 1× every 3 months. On the 6^th^ visit, the children and their mothers were invited back to the Clinic for follow-up examinations and measurement of anthropometric parameters and evaluation of the dietary habits acquired during the dietary education.

### Statistical analysis

Quantitative and rank variables were analysed with non-parametric tests: Mann-Whitney test for comparisons between groups and Wilcoxon signed-rank test for comparisons between two time points (before and after dietary intervention) within one group. Dichotomous variables (yes/no) were compared between groups with Fisher exact test, and between time points with McNemar’s χ2 test. Differences with p<0.05 were considered statistically significant. Statistical analysis was performed with Statistica 13 software.

## Results

### Study group characteristics

Out of the 94 children who qualified for the study, 26 (27.7%) opted out from further participation in education, at various stages. Those who did not complete the study came from families living in rural areas, and their mothers mostly had primary or vocational education. In terms of comparison of anthropometric parameters, the children who completed the dietary intervention did not differ from those who dropped out of the intervention. Sixty-eight children were further analysed. Based on BMI SDS, 59 (86.8%) children were found to be obese and 9 (13.2%) overweight. In 41 children (60,3%) BMI exceeded +3 SDS and in almost one in 10 subjects +6 SDS. Due to the relatively small size of the study population, children with overweight and obesity were combined into one group.

The education levels of the studied children’s mothers varied. Almost half (47.8%) of them had secondary education, more than a third (35.8%) had higher education, and primary or vocational education 8.9% and 7.5% respectively. The vast majority of mothers (83.6%) were professionally active. In the study group, 2/3 (64.2%) of the mothers lived in urban areas and 1/3 (35.8%) in rural areas. Nearly 3/4 (68.7%) of the mothers were also characterized by excessive body weight.

Before the dietary intervention, the relationship between mothers’ nutritional status and the prevalence of overweight and obesity in their children was also analysed. No such relationship was observed (p=0.08). However, it was found that the prevalence of overweight decreased and the prevalence of obesity increased in children following an increase in maternal BMI.

In addition, the BMI of the mothers of children with overweight and obesity was assessed before the dietary intervention. It was found that the BMI of mothers of children with obesity was significantly higher compared to mothers of children with overweight (x̅=28.28 ± 4.98 vs. x̅=23.46 ± 5.71, respectively; p=0.02).

### The effect of one-year dietary intervention on measured anthropometric parameters of the studied children and their mothers

For some measurements, the differences in the number of measured biochemical parameters were caused by insufficient material collected for the assay, haemolysis of the blood and problems related to the consumption of the recommended amount of glucose in the OGTT by the subjects.

One year of dietary education resulted in a significant (p<0.00001) improvement in body weight, waist and hip circumference and WHtR (**Table 1**). There was also a significant increase in the amount of lean muscle tissue and a decrease in the percentage of body fat in the studied children.

**Table 1:** Changes in measured anthropometric parameters in the studied children before and after the dietary intervention.

Measured parameter	n	Before the dietary intervention		After the dietary intervention		Difference	p
		*x̅* ± SD	Me (min.-max.)	*x̅ *± SD	Me (min.-max.)	*x̅* ± SD	
Weight SDS	68	3.47 ± 1.57	3.53 (0.05-7.25)	2.83 ± 1.62	2.57(-0.19-7.94)	−0.64 ± 082	<0.00001
BMI SDS	68	3.70 ± 1.68	3.56 (1.12-9.68)	2.90 ± 1.70	2.76 (0.09-8.57)	−0.80 ± 0.96	<0.00001
Waist circumference SDS	68	4,27 ± 1.79	3.91 (1.60-9.76)	3.50 ± 1.92	3.15 (0.07-9,22)	−0,.77 ± 1.23	<0.00001
Hip circumference SDS	68	2.85 ± 1.37	2.74 (0.77-7.14)	2.07 ± 1.48	1.83 (-0.32-8.38)	−0.78 ± 0.97	<0.00001
WHtR	68	0,60 ± 0.06	0.58 ( 0.50-0.82)	0.56 ± 0.07	0.55 (0.43-0.77)	−0.04 ± 0.04	<0.00001
Adipose tissue [kg]	66*	24.62 ± 12.56	2.22 (6.20-61,60)	22.86 ± 12.23	19.55 (4.20-65.20)	−1.76 ± 4.85	0.03
Adipose tissue percentage	66*	32.27 ± 5.8	33.3 (18.4-42,20)	30.13 ± 7.02	30.05 (12.4-42,20)	−2.14 ± 3.54	<0.00001
Lean muscle tissue [kg]	66*	43.89 ± 4.47	44.6 (18.4-76.30)	45.23 ± 14.01	45.85 (19.80-81.10)	1.34 ± 2.83	<0.00001

BMI, body mass index; WHtR, waist-height ratio; SDS, standard deviation score; SD, standard deviation; p, probability; x̅, mean value; n, sample size; * in 2 children adipose and muscle tissue were not assessed due to their young age (<5).

After dietary intervention, it was additionally shown that the children of mothers with normal BMI were significantly more likely to have reduced body weight than children of mothers with overweight and obesity (p=0.02). The difference in BMI of mothers of children with obesity and mothers of children with overweight after the dietary intervention was further assessed. On average, the BMI of mothers of children with obesity was significantly higher compared to the BMI of mothers of children with overweight (28.9 ± 4.8 vs. 25.4 ± 5.5, respectively; p=0.01).

### Effect of one-year dietary education on measured parameters of lipid metabolism in the studied children

After one year of dietary education, significant improvement in almost all measured parameters of lipid metabolism was observed in the children who completed the education programme, with the exception of total cholesterol concentrations (**Table 2**). There was a significant increase of HDL-cholesterol fraction (p<0.00001) and a significant reduction of LDL-cholesterol and TG fraction (p=0.02).

**Table 2:** Changes in lipid and glucose metabolism parameters before and after the dietary intervention.

Measured parameter	n	Before the intervention	After the intervention	Difference	p
		*x̅* ± SD	*x̅* ± SD	*x̅* ± SD	
Total cholesterol [mg/dl]	67	166.11 ± 27.62	161.91 ± 29.9	−4.20 ± 20.39	0.22
HDL-CH [mg/dl]	67	46.19 ± 10.98	51.5 ± 13.8	5.31 ± 7.72	<0.00001
TG [mg/dl]	67	114.45 ± 60.59	97.5 ± 49.4	−16.95 ± 53.98	0.02
LDL-CH [mg/dl]	66	108.89 ± 26.12	102.6 ± 29.4	−6.29 ± 19.23	0.02
Fasting glucose [mg/dl]	67	88.44 ± 7.87	86.25 ± 8.58	−2.19 ± 9.39	0.04
Glucose in 120’ of OGTT [mg/dl]	65	110.90 ± 20.32	102.80 ± 17.67	−8.10 ± 27.43	0.01
Fasting insulin [µIU/ml]	66	21.37 ± 10.86	16.46 ± 8.35	−4.91 ± 11.56	0.001
Insulin in 120’ of OGTT [µIU/ml]	64	125.02 ± 105.09	86.42 ± 68.19	−38.60 ± 98.08	0.005
HOMA –IR	65	4.74 ± 2.49	3.53 ± 1.87	-1.21 ± 2.72	0.002

HDL, CH-HDL-cholesterol; TG, triglycerides; LDL-CH, LDL-cholesterol; OGTT, oral glucose tolerance test; HOMA-IR, insulin resistance index ;SD, standard deviation; p, probability; x̅, mean value; n, sample size.

### Effect of one-year dietary education on selected parameters of carbohydrate metabolism in the studied children

As shown in **Table 2**, there was a significant effect of annual dietary education on the improvement of all measured carbohydrate parameters in the study group.

### Effect of one-year dietary education on the change in frequency of consumption of selected food groups in the children and their mothers included in the study

Sixty-eight questionnaires from children and 67 questionnaires from mothers before and after the one-year dietary intervention were eligible for detailed analysis. 2 mothers did not agree to complete their dietary questionnaire – one before the intervention and the other after the dietary intervention. Two children did not complete the questionnaires – one regarding the consumption of sweetened dairy products and the other regarding the use of cooking techniques.

The one-year dietary education did not have the same effect on the change in eating habits of children and their mothers (**Table 3**). Mothers significantly increased the number of meals consumed, from three to four per day (p=0.02). The children, on the other hand, significantly decreased the frequency of sweets consumption (p=0.0001). Before the intervention, they consumed sweets most commonly several times a week, while after the intervention they consumed sweets once a week on average.

**Table 3:** Effect of dietary intervention on chosen eating habits in children and their mothers.

Eating habits		R	Children n = 68		Mothers n = 66	
			Before intervention	After intervention	Before intervention	After intervention
			*x̅* ± SD	*x̅* ± SD	*x̅* ± SD	*x̅* ± SD
Number of meals consumed daily	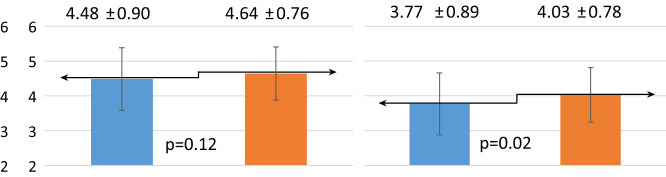					
Frequency of sweets consumption	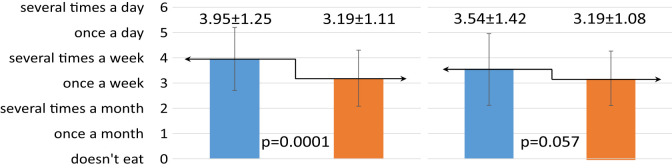					
Frequency of vegetables consumption	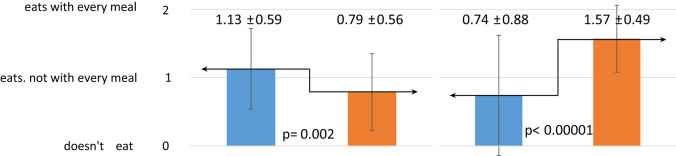					
Frequency of whole greain cereal products consumption	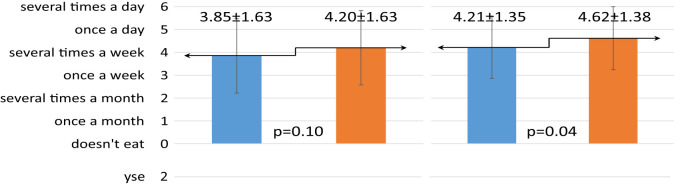					
Self-assesment – do they consider their nutrition correct?	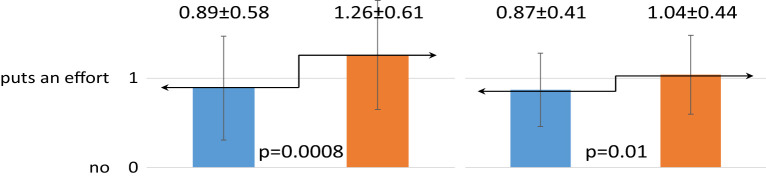					

R, ranks; SD, standard deviation; x̅, mean value; p, probability; n, sample size.

In contrast, the nutritional intervention had a negative effect on the frequency of children’s vegetable consumption. After the intervention, children consumed vegetables significantly less often (p=0.002). Mothers, on the other hand, significantly increased the frequency of their consumption (p<0.00001) and ate them with every meal of the day. The mothers also significantly improved the frequency of consumption of whole grain cereal products, up to once per day (p=0.04).

In addition, the dietary intervention resulted in a significant improvement in children’s and their mothers’ self-assessment of correct dietary habits (p=0.0008; p=0.01, respectively). However, there was no effect of the dietary intervention on changing the frequency of fruit, fish and fast-foods consumption by either the children or their mothers.

As seen in **Table 4**, the dietary intervention resulted in a more frequent change in the assessed eating habits in children than in their mothers. There was a significant 17.7% reduction in the frequency of snacking between meals (p=0.009) and a significant 20.6% reduction in the frequency of after-dinner snacking in the children studied (p=0.007). The educational meetings significantly influenced the water drinking habit, primarily in children. There was a significant 20.6% increase in the frequency of water consumption in the children studied (p=0.003). The nutritional intervention also influenced the resignation from the consumption of sweet drinks, not only by the children, but also by their mothers. A 26.5% significant reduction in the frequency of sweetened beverage consumption was observed in the studied children (p=0.0002) and a 13.6% significant reduction in the frequency of sweetened beverage consumption (p=0.03) in the studied mothers. The dietary intervention resulted in a positive change in the acceptance of vegetables by children, but not by mothers. There was a 70.5% significant increase in the frequency of vegetable acceptance in children, while there was a 10.6% significant decrease in mothers. (p=0,02). Unfortunately, acceptance of vegetable intake did not result in realistically higher vegetable intake. Furthermore, there was no effect of the dietary intervention on the other dietary habits assessed, such as first and second breakfast consumption, and acceptance of fruit. **Table 5** shows the effect of one year of dietary education on the consumption preferences of selected dairy products. The dietary intervention significantly increased the consumption of plain dairy products by the study children, but not by their mothers. A significant 27.9% increase in the frequency of consumption of plain dairy products (p=0.005) by the studied children was observed. Moreover, the effect of the one-year intervention was a significant 26.9% reduction in the frequency of consumption of sweetened dairy products by the children (p=0.001). The opposite effect of the dietary intervention was observed in mothers. There was a 24.3% significant increase in the frequency of mothers’ consumption of sweetened dairy products (p=0.001). On the other hand, no significant effect of the dietary intervention was observed on the consumption of milk by the studied children. Half of the children did not consume milk both before and after the one-year dietary intervention.

**Table 4:** Effect of one-year dietary intervention on changes in dietary habits in studied children and their mothers.

Assesed habit		Assessed habit – before intervention							
		Children n = 68				Mothers n = 66			
		No	Yes	Total	p	No	Yes	Total	p
		n (%)	n (%)	n (%)		n (%)	n (%)	n (%)	
Assessed habit – after intervention	Do they snack between meals								
	No	0 (0%)	15(22.1%)	15 (22.1%)	0.009	1 (1.5%)	3 (4.5%)	4 (6%)	0.61
	Yes	3 (4.4%)	50 (73.5%)	53(77.9%)		1 (1.5%)	61 (92.5%)	62 (94%)	
	Total	3 (4.4%)	65(95.6%)	68 (100%)		2 (3.0%)	64 (97.0%)	66 (100%)	
	Do they snack after dinner								
	No	29(42.7%)	19(27.9%)	48 (70.6%)	0.007	25 (37.9%)	14 (21,2%)	39(59.1%)	0.06
	Yes	5 (7.3%)	15 (22.1%)	20(29.4%)		5 (7.6%)	22 (33,3%)	27(40.9%)	
	Total	34(50.0%)	34(50.0%)	68 (100%)		30 (45.5%)	36 (54,5%)	66 (100%)	
	Do they like vegetables								
	No	4 (5.9%)	4 (5.9%)	8 (11.8%)	<0.00001	1 (1.5%)	7 (10.6%)	8 (12.1%)	0.02
	Yes	52(76.4%)	8 (11.8%)	60(88.2%)		0 (0%)	58 (87.9%)	58(87.9%)	
	Total	56(82.3%)	12(17.7%)	68 (100%)		1(1.5%)	65(98.5%)	66 (100%)	
	Do they drink water								
	No	4 (5.9%)	3 (4.4%)	7 (10.3%)	0.003	7 (10.6%)	4 (6.1%)	11(1.,7%)	0.75
	Yes	17 (25%)	44 (64.7%)	61(89.7%)		6 (9.1%)	49 (74.2%)	55(83.3%)	
	Total	21(30.9%)	47(69.1%)	68 (100%)		13 (19.7%)	53 (80.3%)	66 (100%)	
	Do they drink sweet beverages								
	No	10 (14.8%)	20(29.4%)	30 (44.2%)	0.0002	14 (21.2%)	12(18.2%)	26(39.4%)	0.03
	Yes	2 (2.9%)	36 (52.9%)	38(55.8%)		3 (4.6%)	37 (56.1%)	40(60.6%)	
	Total	12 (17.7%)	56(82.3%)	68 (100%)		17 (25.8%)	49(74.2%)	66 (100%)	

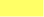
– habit habit declared before intervention, 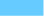
– habit seen only before intervention, 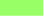
– habit declared after intervention, 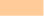
– habit seen only after intervention, p- probability, n – sample size.

**Table 5:** Effect of one-year dietary intervention on consumption of selected dairy products consumed by studied children and their mothers.

Assesed habit		Before intervention – dairy products consumed daily							
		Children n = 68				Mothers n = 66			
		No	Yes	Total	p	No	Yes	Total	P
		n (%)	n (%)	n (%)		n (%)	n (%)	n (%)	
After intervention- dairy products consumed daily	Plain dairy products								
	No	30 (44.1%)	4 (5.9%)	34 (50.0%)	0,005	11(16.7%)	16 (24.2%)	27 (40.9%)	1.0
	Yes	23(33.8%)	11 (16.2%)	34(50.0%)		15(22.7%)	24 (36.4%)	39 (59.1%)	
	Total	53 (77.9%)	15(22.1%)	68 (100%)		26(39.4%)	40 (60.6%)	66 (100%)	
		Children n=67				Mothers n=66			
	Sweetened dairy products								
	No	19 (28.3%)	23(34.4%)	42 (62.7%)	0,001	44(66.7%)	3 (4.5%)	47 (71.2%)	0.001
	Yes	5 (7.5%)	20 (2.,8%)	25(37.3%)		19(28.8%)	0 (0%)	19(28.8%)	
	Total	24 (35.8%)	43(64.2%)	67 (100%)		63(95.5%)	3 (4.5%)	66 (100%)	

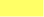
– habit declared before intervention, 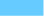
– habit seen only before intervention, 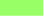
– habit declared after intervention, 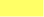
– habit seen only after intervention, p – probability, n – sample size.

Another diet component assessed was cereal products. As shown in **Table 6**, the one-year dietary intervention had a significant effect of reducing sweetened breakfast cereal by 36.8% in children (p=0.00003). However, no such effect was observed in mothers.

**Table 6:** Effect of one-year dietary intervention on cereal products consumption in studied children and their mothers.

Assessed habit		Before intervention – cereal product consumed daily							
		Children n = 68				Mothers n = 66			
		No	Yes	Total	p	No	Yes	Total	P
		n (%)	n (%)	n (%)		n (%)	n (%)	n (%)	
After intervention – cereal product consumed daily	Oat flakes								
	No	34 (50.0%)	7 (10.3%)	41 (60.3%)	0.02	33 (50%)	5 (7.5%)	38 (57.5%)	0.21
	Yes	20(29.4%)	7 (10.3%)	27(39.7%)		11 (17%)	17 (25.5%)	28 (42.5%)	
	Total	54 (79.4%)	14 (20.6%)	68 (100%)		44 (67%)	22 (33%)	66 (100%)	
	Sweetened breakfast cereal								
	No	23 (33.8%)	29 (42.7%)	52 (76.5%)	0.00003	48(72.7%)	5 (7.6%)	53 (80.3%)	1.0
	Yes	4 (5.9%)	12 (17.6%)	16(23.5%)		4 (6.1%)	9 (13.6%)	13 (19.7%)	
	Total	27 (39.7%)	41 (60.3%)	68 (100%)		52 (78.8%)	14 (21.2%)	66 (100%)	
	Wheat bread								
	No	25 (36.8%)	23 (33.8%)	48 (70.6%)	0.001	34 (51.5%)	13 (19.7%)	47 (71.2%)	0.26
	Yes	5 (7.3%)	15 (22.1%)	20(29.4%)		7 (10,.%)	12 (18.2%)	19 (28.8%)	
	Total	30 (44.1%)	38 (55.9%)	68 (100%)		41 (62.1%)	25 (37.9%)	66 (100%)	
	Wheat pasta								
	No	18 (26.5%)	20 (29.4%)	38 (55.9%)	0.02	19 (28.8%)	16 (24.2%)	35 (53.0%)	0.32
	Yes	7 (10.3%)	23 (33.8%)	30(44.1%)		10 (15.2%)	21 (31.8%)	31 (47.0%)	
	Total	25 (36.8%)	43 (63.2%)	68 (100%)		29 (44.0%)	37 (56.0%)	66 (100%)	
	Wholemeal pasta								
	No	38 (55.9%)	5 (7.3%)	43 (63.2%)	0.002	41 (62.1%)	5 (7.6%)	46 (69.7%)	0.06
	Yes	22(32.3%)	3 (4.5%)	25(36.8%)		14 (21.2%)	6 (9.1%)	20 (30.3%)	
	Total	60 (88.2%)	8 (11.8%)	68 (100%)		55 (83.3%)	11 (16.7%)	66 (100%)	
	Brown rice								
	No	37 (54.4%)	6 (8.8%)	43 (63.2%)	0.002	40 (60.6%)	4 (6.1%)	44 (66.7%)	0.02
	Yes	23(33.8%)	2 (3.0%)	25(36.7%)		15(22.7%)	7 (10.6%)	22(33.3%)	
	Total	60 (88.2%)	8 (11.7%)	68 (100%)		55 (83.3%)	11(16.7%)	66 (100%)	

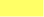
– habit declared before intervention, 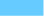
– habit seen only before intervention, 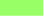
– habit declared after intervention, 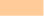
– habit seen only after intervention, p – probability, n – sample size.

Dietary education significantly reduced the frequency of children’s consumption of wheat bread by 26.5% (p=0.001), but had no effect on increasing the consumption of whole-grain bread. No effect was observed in mothers. One-year dietary education significantly influenced the replacement of wheat pasta with wholemeal pasta in the studied children. There was a 19.1% significant decrease in the frequency of wheat pasta consumption (p=0.02) in favour of a 25.0% significant increase in the frequency of wholemeal pasta consumption (p=0.002). Additionally, dietary education significantly increased the frequency of brown rice consumption by 25.0% in children (p=0.002) and by 16.6% in mothers (p=0.02). No such effect was observed for white rice and buckwheat kasha consumption.

The effect of the one year dietary intervention on the type of meat consumed was also analyzed (**Table 7**). The dietary intervention significantly, by 16.1%, reduced the frequency of children’s consumption of pork meat (p=0.02). For the other two assessed meat varieties and for the three assessed types of meat consumed by the mothers, no such effect was observed.

**Table 7:** Effect of one-year dietary education on the types of meat consumed by studied children and their mothers.

Assessed habit		Before intervention – type of meat consumed							
		Children n = 68				Mothers n = 66			
		No	Yes	Total	p	No	Yes	Total	p
		n (%)	n (%)	n (%)		n (%)	n (%)	n (%)	
After intervention – type of meat consumed	Pork								
	No	31 (45.6%)	15 (22.0%)	46 (67.6%)	0.02	25(37.9%)	13(19.7%)	38(57.6%)	0.38
	Yes	4 (5.9%)	18 (26.5%)	22 (32.4%)		8 (12.1%)	20(30.3%)	28(42.4%)	
	Total	35 (51.5%)	33 (48.5%)	68 (100%)		33(50.0%)	33(50.0%)	66 (100%)	
	Beef								
	No	51 (75.0%)	7 (10.4%)	58 (85.4%)	0.77	39(59.1%)	8 (12.1%)	47(71.2%)	0.80
	Yes	5 (7.3%)	5 (7.3%)	10 (14.6%)		8 (12.1%)	11(16.7%)	19(28.8%)	
	Total	56 (82.3%)	12 (17.7%)	68 (100%)		47(71.2%)	19(28.8%)	66 (100%)	

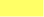
– habit declared before intervention, 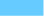
– habit seen only before intervention, 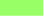
– habit declared after intervention, 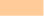
– habit seen only after intervention, p – probability, n – sample size.

The last food group analysed was the type of fat and the preferred cooking techniques used. As shown in **Table 8**, one year’s dietary education had a notable effect only on mothers.

**Table 8:** Effect of one-year dietary intervention on cooking techniques and type of cooking fats used by studied children and their mothers. .

Assessed habit		Children n = 67				Mothers n = 66			
		No	Yes	Total	p	No	Yes	Total	p
		n (%)	n (%)	n (%)		n (%)	n (%)	n (%)	
After intervention – cooking technique used and type of fat chosen	Cooking, steaming, grilling								
	No	3 (4.5%)	6 (8.9%)	9 (13.4%)	0.60	6 (9.1%)	1 (1.5%)	7 (10.6%)	0.001
	Yes	9 (13.4%)	49 (73.2%)	58 (86.6%)		14(21.2%)	45 (68.2%)	59(89.4%)	
	Total	12 (17.9%)	55 (82.1%)	67 (100%)		20 (30.3%)	46(69.7%)	66 (100%)	
	Plant-based								
	No	2 (3.0%)	8 (11.9%)	10 (14.9%)	0.38	4 (6.1%)	2 (3.0%)	6 (9.1%)	0.01
	Yes	13 (19.4%)	44 (65.7%)	57 (85.1%)		12(18.2%)	48 (72.7%)	60(90.9%)	
	Total	15 (22.4%)	52 (77.6)	67 (100%)		16 (24.3%)	50(75.7%)	66 (100%)	

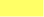
– habit declared before intervention, 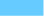
– habit seen only before intervention, 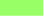
– habit declared after intervention, 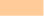
– habit seen only after intervention, p – probability, n – sample size.

There was a 19.7% significant increase in the frequency of fat-free cooking techniques used (p=0.001) and a 15.2% significant increase in the frequency of use of plant-based fat by the mothers surveyed (p=0.01).

## Discussion

Overweight and obesity in the developmental age population remains an unresolved problem that is increasing worldwide (36). It is necessary to take measures to prevent new and eliminate existing disorders. One such action is a change in lifestyle, an important component of which, alongside physical activity, is a change in eating habits. Sisson et al. (37), in a systematic review of interventions conducted in children with obesity, showed that out of 45 dietary interventions, 87.0% resulted in the desired outcome.

The dietary intervention we conducted was based on the principles of healthy nutrition and the 2009 ‘Healthy Food Pyramid’ model developed by experts from the Institute of Food and Nutrition (24). In addition, it was conducted in the presence of one of the caregivers – the mother. These efforts showed a significant effect on improving both somatic development parameters, biochemical indices and nutritional habits in the studied children.

One year of dietary education in the studied children resulted in significant differences in the measured parameters of somatic development (respectively: BMI SDS −0.80; waist circumference SDS −0.77; WHtR −0.04; −1.76 kg body fat; −2.14 body fat percentage and +1.34 kg lean muscle tissue). The effects obtained in our study may be due to several reasons including the fact that each educational activity took place in the presence of one of the parents. In a similar study, called WATCH IT (38), researchers found smaller differences in reduction of waist circumference, −0.08 SDS. In contrast, unfavourable results were observed for BMI SDS and body fat percentage, (by +0.03 SDS BMI and +1.4% body fat, respectively). The cited authors explain this result, among other things, by not being able to involve the child’s caregiver in the study. Conversely, in the study by Savoye et al. (39), a 4.0% reduction in body fat (−3.7kg) was achieved. Greater therapeutic success in these studies was achieved by involving families who attended meetings with a dietician and physical activity specialist. In a study evaluating one of the risk factors for cardiovascular disease, expressed as the ratio of waist circumference to body height in a group of 5–12 and 13–17 year-olds, Ranucci et al. (40) obtained a significant reduction in WHtR, from 0.63 to 0.61, in a group of 5-12 year-old children, and from 0.65 to 0.63 in a group of adolescents. In the above studies, active physical activity was an additional intervention. It is known from the literature that moderate exercise alone does not cause weight loss, but when combined with changes in dietary habits, significant weight loss can be achieved and maintained. Therefore, one element of education in our study was to encourage children and their parents to undertake additional physical activity. However, at the end of the one-year dietary intervention, no significant increase in time spent on additional physical activity was observed. In our study, children with excessive body weight as a result of dietary education and with maternal involvement achieved significantly greater differences in measured parameters of somatic development than in the work presented above. The available literature shows that parents, especially mothers, participate in the formation of the child’s eating habits by providing appropriate products in the diet that they consider healthy. The mother is also a role model and controls food intake (41). A Cochrane review of erspectiv clinical trials of children in two age groups, 6–11 and 12–17 years, on “Interventions for the treatment of childhood obesity”, found that behavioural interventions related to lifestyle changes, combined with parental involvement, are most effective (42). These interventions have been called the “gold standard for the management of childhood obesity” (43). Moreover, in our study, significant improvements were observed in body composition, not only in terms of fat reduction, but also in the percentage of muscle mass. The results obtained appear to be significant, as in some studies a reduction in muscle mass has also been reported in parallel with a reduction in fat mass (44). A reduction in lean body mass results in a “reduced metabolic rate” and consequently “slower weight loss” (45). In addition, it has been shown that the type of dairy consumed plays an important role in the prevention of overweight and abdominal obesity. In a study by Bradllee et al. (46), evaluating the association between intake of different foods and abdominal obesity in children and adolescents, conducted as part of NHANES III, the cited authors showed that dairy intake presents a negative correlation with central obesity. An identical association, but only in a group of girls, was found by Abreu et al. (47) and in a cross-sectional study by Hirschler et al. (48). This is explained by the protective role of calcium, which, through its effect on the regulation of energy metabolism, reduces lipogenesis in adipocytes and increases both faecal fat excretion and fat oxidation. In addition, whey proteins contained in dairy products cause a greater feeling of satiety (49) and are characterized by their high content of branched-chain amino acids, which are involved in muscle protein synthesis. Therefore, the energy consumed with dairy products is used to build muscle mass, at the expense of body fat (50). In our study, a significant increase in dairy consumption in the form of plain yoghurt after a one-year dietary intervention may have contributed to this outcome.

The changes in anthropometric parameters found in our study were accompanied by changes in measured biochemical parameters. The one-year dietary intervention significantly improved the lipid profile and the carbohydrate metabolism parameters.

The one-year dietary education applied in our study resulted in a significant reduction in LDL-cholesterol (by 6.29 mg/dl), triglycerides (by 16.95 mg/dl) and a significant increase in HDL-cholesterol (by 5.31 mg/dl). There was also a significant effect of the dietary intervention on the reduction of all measured parameters of carbohydrate metabolism, i.e.: fasting glucose and insulin levels (by 2.19 mg/dl and 4.91 µIU/ml, respectively) and in the 2h point of OGTT (glucose by 8.10 mg/dl and insulin by 38.60 µIU/ml). This effect may be related to both an improvement in anthropometric parameters and a change in dietary habits. The link between changes in BMI SDS and cardiovascular system and body composition has been analysed in several studies (51, 52). These publications noted that a reduction of 0.25 BMI SDS could be considered clinically significant for improving fasting insulin sensitivity and improving the total cholesterol/HDL-cholesterol ratio. However, greater benefits were observed with a reduction of 0.5 in BMI SD (53), and reduced markers of insulin resistance were indeed found when an even more significant reduction in BMI (≥0.5 BMI SDS) was achieved (54). In our study, after a one-year dietary intervention, the change in SDS BMI was −0.80. This change may therefore have contributed to an improved lipid profile and reduced insulin resistance.

As mentioned, diet is directly and indirectly related to cardiovascular risk factors, so improvements in lipid metabolism parameters depend not only on reducing body weight and body fat percentage, but also on diet composition (55). In the current study, the reduction in LDL-cholesterol levels may have been further associated with a reduction in pork consumption during the one-year dietary intervention. In contrast, the reduction in triglycerides may have been associated with significantly lower sweets intake and more frequent choice of fat-free cooking techniques. In a study by Wengle et al. (56), a change in dietary habits through an increase in the consumption of whole-grain bread and fruit and vegetables was associated with a reduction in LDL-cholesterol levels. In our study, children significantly increased their intake of whole-grain cereal products after a one-year dietary intervention. In contrast, other researchers attributed the improvement in lipid and carbohydrate parameters not only to an increase in intake of whole-grain cereal products, but also to a reduction in fast-foods consumption. Gingras et al. (57) showed that consumption of fast food less than 1× per week was associated with less severe obesity in girls and less insulin resistance in boys. In our one-year dietary intervention, children’s fast-food intake did not change much and they consumed these foods with similar frequency (on average 1 time per month), both before and after the one-year dietary intervention. Investigating the association of different dietary patterns with the prevalence of insulin resistance in children, Karatzi et al. (58) found that increased consumption of margarine, sweets and salty snacks was positively correlated with insulin resistance, while breakfast consumption showed a negative correlation. In our study, the one-year dietary intervention did not result in a significant change in the habit of eating the first breakfast. Before the dietary intervention, 60 (88.2%) and after the intervention 66 (97.1%) children consumed first breakfast. The improvement in measured parameters of carbohydrate metabolism can therefore be attributed to a significant improvement in other dietary habits, particularly the elimination of the consumption of sweets, sugar-sweetened beverages, sweetened, flavoured dairy products and sweetened, flavoured cereals, and the inclusion of plain yoghurt, oatmeal, brown rice and wholemeal pasta into the diet. Due to their favourable composition, i.e. the predominance of complex carbohydrates over simple carbohydrates, their consumption is followed by a gradual increase in blood glucose concentration, which ‘‘slows down” and reduces insulin secretion, preventing significant glycaemic fluctuations, prolonging the feeling of satiety and reducing the desire to snack between meals. As a matter of fact, in the dietary intervention we carried out, the children significantly reduced snacking between meals and after dinner. Our results are similar to those obtained by other authors with a low glycaemic index diet (59, 60). These data may indicate that proper dietary management, based on healthy eating principles, is important, and that the use of a dietary pattern such as the “Healthy Food Pyramid” (24) significantly improved the metabolic profile of the children studied. Meta-analyses (61) on dietary treatment of people at increased risk of cardiovascular disease and the effect of dietary treatment on lipid and carbohydrate metabolism are mainly concerned with adults. Few papers are devoted to the developmental age population. Therefore, the results we obtained can be used to develop dietary recommendations for this population. In addition, the reduction of disease risk factors in children in our study has major clinical implications. The improvements in lipid and carbohydrate metabolism in the present study are therefore as significant as those that can be achieved with pharmacological treatment, but without the fear of adverse effects from the medications used (62).

Analysing the dietary habits of children and their mothers in our studied population after a one-year dietary intervention showed that dietary education did not affect all children and their caregivers in the same way. Greater change in abnormal eating behaviour was observed only in children. An explanation for this could be sought, for example, in the way the intervention was conducted. This is because the children were actively involved in the training, kept food diaries which were analysed together and any errors were modified on an ongoing basis, which further enables the children to take control of their eating habits. In contrast, parental feeding errors and their replication are most often the result of parents’ reluctance to change their own personal attitudes. The effectiveness of treatment of a child with excessive body weight is therefore increased if their parents also decide to change their diet. In the current study, it appears that only a small number of mothers’ eating habits was modified by appropriate health-promoting education. This modification only addressed the correct number of meals consumed per day, more frequent consumption of vegetables, whole grain cereal products including brown rice, drinking sugary drinks, using fat-free cooking techniques and consuming vegetable fats.

Regardless of how the dietary modification plan is formulated, what tools are used and how long we plan to intervene, in the case of children it is important that their parents are also involved in the programme. This is because a child has limited capacity to make changes, such as eating habits. Numerous studies also indicate a lack of understanding on the part of parents on both how to form proper eating habits in their children and how to implement these recommendations. Therefore, a reasonable suggestion would be to provide parents with suitable education programmes, with opportunities for extended contact with nutrition professionals (63).

The study we conducted had some limitations. The lack of a control group made it impossible to compare the effect and strength of the interventions undertaken. On the other hand, all children, both with overweight and normal weight, and their families should have the same opportunity to obtain correct information on proper nutrition and current guidelines. Another limitation may be the size of the sample population that was subjected to the intervention. This is because the intervention was only targeted at the group of children referred to the Clinic for diagnosis of the causes of obesity. Therefore, this group may not be fully representative of all children with obesity, as children seeking treatment were more motivated to reduce their weight. Furthermore, the size of the group was limited, due to the time-consuming form of individual education. Additionally, due to the small number of children reaching the study’s endpoint, the group was not divided by sex and age for statistic analysis. During the 12-month dietary intervention, 26 (27.7%) participants dropped out at various stages of the intervention. The manner in which the questionnaires were completed and the under- or overestimation of foods that may be relevant to the prevention and treatment of excess body weight also need to be considered, as underestimation of food intake is a major problem in childhood obesity research.

In spite of the above limitations, it has been shown that educational measures aimed at family-based change in dietary behaviour have a positive effect on improving measured biochemical parameters. The plethora of health education methods means that an optimal model is constantly being pursued that will, in a meaningful way, help patients to sustainably accept the information provided. In the presented study, a simple model of the Healthy Eating Pyramid (24) was used, supported by the principles of healthy eating. As a result of its application, many children achieved not only a change in eating habits, but also an improvement in measured anthropometric and biochemical parameters without the use of pharmacological intervention.

However, further research is needed to assess the cause and effect of the nutritional interventions that were carried out.

## Conclusions

1. In the studied group of children, the one-year dietary intervention contributed to a significant reduction in body weight, waist and hip circumference and body fat percentage. Moreover, a significant improvement in the measured parameters of carbohydrate metabolism, lipid metabolism, with the exception of total cholesterol concentration, was shown.
2. The applied nutrition pattern, based on the Healthy Food Pyramid, is an effective tool for eliminating or at least reducing incorrect eating habits in children with excess body weight.
As a result of the educational activities carried out, children significantly reduced their consumption of sweets and sugary drinks, sweetened dairy products and sugary breakfast cereals. They also reduced their consumption of wheat pasta and bread and their consumption of pork meat. They also significantly reduced “snacking” between meals and after dinner. However, they significantly increased their intake of water, natural dairy products and whole grain cereals.
3. The value of longitudinal dietary education, with constant monitoring of the effectiveness of the diet and the full involvement of the family, especially the mothers of children with excess body weight, was also demonstrated.

## Data availability statement

The raw data supporting the conclusions of this article will be made available by the authors, without undue reservation.

## Ethics statement

The studies involving human participants were reviewed and approved by Bioethics Committee at the Pomeranian Medical University in Szczecin decision number KB-0012/34/11, dated 16th May 2011. Written informed consent to participate in this study was provided by the participants’ legal guardian/next of kin.

## Author contributions

Conceptualization, KSt and MW; Methodology KSt, AH-J, KSa, EP, and MW; Visualization KSt, TJ, JS-D, KSa, EP, and MW; Writing—original draft preparation, KSt, AH-J, TJ, and MW; Writing—review and editing, KSt, AH-J, TJ, JS-D, and MW; Project administration, MW; Supervision, MW; All authors have read and agreed to the published version of the manuscript.

## Conflict of interest

The authors declare that the research was conducted in the absence of any commercial or financial relationships that could be construed as a potential conflict of interest.

The handling editor AMG declared a past co-authorship with the author EP.

## Publisher’s note

All claims expressed in this article are solely those of the authors and do not necessarily represent those of their affiliated organizations, or those of the publisher, the editors and the reviewers. Any product that may be evaluated in this article, or claim that may be made by its manufacturer, is not guaranteed or endorsed by the publisher.
